# Behavioral phenotype of Noonan-like syndrome with loose anagen hair

**DOI:** 10.1192/j.eurpsy.2022.1172

**Published:** 2022-09-01

**Authors:** N. Bouayed Abdelmoula

**Affiliations:** Medical University of Sfax, Genomics Of Signalopathies At The Service Of Medicine, Sfax, Tunisia

**Keywords:** Noonan-like syndrome with loose anagen hair, RAS-MAPK, attention deficit-hyperactivity disorder

## Abstract

**Introduction:**

Noonan-like syndrome with loose anagen hair (NSLH MIM 607721) is associated to mutations in PTPN11, RAF1, BRAF and SHOC2 genes.

**Objectives:**

Here, we report behavioral phenotype of a child suspected to have NSLH.

**Methods:**

A 2-years-old Tunisian child harboring severe pulmonic valvular stenosis was referred to our genetic counselling for genetic assessment. Medical dysmorphology, cytogenetic analysis as well as genetic exploration of RAS-MAPK pathway genes were conducted.

**Results:**

The child had short stature and ectodermal features including ichthyotic skin and thin-soft nails. He has specific hair appearance associated to NS features. In fact, he had a small nasal tip, thick lips and sticking-out rotated ears. He harbored typical nasal voice and loose anagen hair with ungrowing thin hair, sparse and pale scalp hair and eyebrows. He showed cognitive deficits with mental retardation and hyperactive behavior. Considered as having NSLH, cytogenetic analysis revealed a 46,XY formula, but molecular screening of PTPN11, RAF1, BRAF, RIT1 and SHOC2 genes was negative.

**Conclusions:**

Mutations within the RAS‐MAPK signaling pathway affect neurophysiologic activity in brain regions underlying attention and executive functions. Children with rasopathies demonstrated higher rates of attention deficit-hyperactivity (ADHD) and autism spectrum disorders. However, no studies have examined specifically the aspects of behavioral attention in the various types of Rasopathies. A recent study demonstrated that ADHD seems to be higher in children with NSLH and SHOC2 mutation, which is the case of our patient. We suggest that assessment of inattentive and hyperactivity symptoms in children should consider Rasopathies with specific molecular screening.

**Disclosure:**

No significant relationships.

